# Cost of radiofrequency ablation for chronic venous insufficiency: a pilot study of 9 cases at a teaching hospital in Brazil

**DOI:** 10.1590/1677-5449.202401722

**Published:** 2025-07-07

**Authors:** Ana Luiza Carvalho Sartoreli, Henrique Capistrano dos Santos, Thais Keltke Santos Felippe, Júlio César Souza Diniz, Luiz Baldini, Gustavo Muçouçah Sampaio Brandão, Michel Nasser

**Affiliations:** 1 Universidade Federal de São Carlos – UFSCar, São Carlos, SP, Brasil.; 2 Universidade Federal de São Carlos – UFSCar, Hospital Universitário – HU, São Carlos, SP, Brasil.; 3 Hospital dos Fornecedores de Cana de Piracicaba – HFC, Piracicaba, SP, Brasil.

**Keywords:** chronic venous disease, varicose veins, saphenectomy, radiofrequency ablation, cost-effectiveness, teaching hospital, doença venosa crônica, varizes, safenectomia, ablação por radiofrequência, custo-efetividade, hospital universitário

## Abstract

**Background:**

While radiofrequency ablation (RFA) is increasingly used to treat saphenous vein incompetence, its adoption in Brazil may have been hindered by the lack of evidence demonstrating sufficient added value to justify its cost-effectiveness.

**Objectives:**

To perform RFA in 9 patients with lower extremity varicose veins and determine the procedural costs per patient at a Brazilian teaching hospital.

**Methods:**

Nine single-use RFA catheters were purchased by the teaching hospital affiliated with our institution and used in this pilot study. Direct and indirect costs were calculated as sums of the respective cost components of the procedure based on values from the federal government’s price panel. To illustrate the potential cost-effectiveness of RFA, these costs were compared to those of 9 saphenectomy procedures performed on the same day as the RFA procedures. All analyses were descriptive, with no formal statistical testing.

**Results:**

The mean operating room hourly rate for RFA was 127.50 BRL. The costs of anesthetics/medications, materials, and single-use catheter per patient were 32.63 BRL, 81.49 BRL, and 1600.00 BRL, respectively. Patients were absent from work for < 15 days (mean, 11.44 days), not incurring sick leave payments from the Social Security Administration. The total mean cost for RFA was lower than that of same-day saphenectomy (1841.62 BRL vs 2045.40 BRL).

**Conclusions:**

This pilot study provided essential insights into resource utilization at a Brazilian teaching hospital, with the goal of improving treatment efficiency and ensuring the best cost-benefit ratio for patients.

## Resumo

**Contexto:**

Embora a ablação por radiofrequência (ARF) seja cada vez mais utilizada no tratamento de insuficiência da veia safena, sua adoção no Brasil pode ter sido dificultada pela falta de evidências que demonstrem um valor agregado suficiente para justificar sua relação custo-efetividade.

**Objetivos:**

Avaliar a ARF em nove pacientes com varizes de membros inferiores e determinar os custos do procedimento por paciente em um hospital universitário brasileiro.

**Métodos:**

Nove cateteres de ARF de uso único adquiridos pelo hospital universitário afiliado à nossa instituição foram utilizados neste estudo piloto. Os custos diretos e indiretos foram calculados como somas dos respectivos componentes de custo do procedimento, segundo valores do painel de preços do governo federal. Para ilustrar o potencial custo-benefício da ARF, esses custos foram comparados aos de nove safenectomias realizadas no mesmo dia das ARFs. As análises foram descritivas, sem aplicação de testes estatísticos formais.

**Resultados:**

A taxa horária média da sala cirúrgica para ARF foi de R$ 127,50. Os custos com anestésicos/medicamentos, materiais e cateter por paciente foram de R$ 32,63, R$ 81,49 e R$ 1.600,00, respectivamente. Os pacientes se ausentaram do trabalho por < 15 dias (média: 11,44 dias), não incorrendo em pagamentos de licença médica pelo Instituto Nacional do Seguro Social. O custo médio total da ARF foi menor do que o da safenectomia no mesmo dia (R$ 1.841,62 vs. R$ 2.045,40).

**Conclusões:**

Este estudo piloto forneceu dados essenciais sobre a utilização de recursos em um hospital universitário brasileiro, com o objetivo de melhorar a eficiência do tratamento e garantir a melhor relação custo-benefício para os pacientes.

## INTRODUCTION

Chronic venous disease (CVD) is a prevalent condition characterized by chronic venous hypertension, typically resulting from valve incompetence and/or venous outflow obstruction.^1^ CVD encompasses a range of manifestations, including telangiectasias, reticular veins, and varicose veins, which can cause significant patient discomfort and complications. These complications include pain (often described as heaviness), lipodermatosclerosis, atrophie blanche, hyperpigmentation, stasis dermatitis, venous eczema, edema, and ulceration.^2-4^ The global impact of CVD is substantial, with a prevalence as high as 83.6% reported by the Vein Consult Program survey.^5^ In Brazil, an epidemiological study of 1775 patients conducted in Botucatu, a city in the state of São Paulo, estimated that 35.5% of adults have varicose veins, and 1.5% experience severe chronic venous insufficiency with active venous ulcers or scars from previous ulcers.^2^

Vein ligation and stripping has been a standard treatment for CVD in Brazilian teaching hospitals. While this approach can yield satisfactory medium- and long-term results, it also presents drawbacks such as invasiveness, the need for spinal anesthesia, a prolonged recovery period (approximately 30 days), and the use of major hospital resources.^2,6,7^ Endovenous modalities, such as radiofrequency ablation (RFA), offer a minimally invasive alternative to traditional surgery for treating saphenous vein incompetence.^8^ RFA is a single-operator procedure that uses RF-generated thermal energy to occlude the incompetent vein, with advantages such as faster recovery, allowing patients to return to work within 3 to 5 days, and reduced short-term morbidity.^8-11^

While evidence suggests that RFA offers benefits such as reduced postoperative pain, improved symptoms, and reduced operative time compared with traditional surgery,^1,10,12,13^ the perception that it may be more expensive has hindered its widespread adoption.^9^ In particular, some public health settings in Brazil may have been hesitant to implement RFA due to the lack of evidence demonstrating sufficient added value to justify its cost-effectiveness, especially given the potential impact on health system budgets.^14^

This pilot study aimed to address this concern by analyzing the procedural costs per patient undergoing RFA for lower extremity varicose veins at a teaching hospital in Southeast Brazil. The preliminary data generated from this study will provide hospital decision-makers and medical staff with valuable insights to guide resource allocation, improve treatment efficiency, and ultimately ensure the best cost-benefit ratio for patients.

## METHODS

This pilot study was conducted at the teaching hospital affiliated with our institution, located in São Carlos, a city in the state of São Paulo, Brazil. The study was approved by the institution’s Research Ethics Committee (opinion number: 5.572.132; approval number: CAAE 59105522.0.0000.5504). Written informed consent was obtained from each participant prior to inclusion in the study. The study was reported according to the Consolidated Health Economic Evaluation Reporting Standards 2022 (CHEERS 2022) Statement.

Our institution purchased 9 single-use RFA catheters (ClosureFast™ RFA System, Medtronic, Inc, Minneapolis, MN, USA) and made them available for treatment of lower extremity varicose veins at the vascular surgery outpatient clinic from May to September 2023. Eligible participants were all patients aged ≥ 18 years with an American Society of Anesthesiologists (ASA) preoperative physical status of 2, a 7–12 mm saphenous vein diameter, and C2–C5 Clinical, Etiological, Anatomical, Pathophysiological (CEAP) clinical class. Patients with phlebitis, previous varicose vein surgery, sclerotherapy, or active venous ulcers (C6) were excluded.

Our patient selection was dictated by the limited availability of RFA catheters; only 9 units were accessible for this study. Consequently, we included only patients who were both appropriate candidates for the procedure and treatable with the catheters provided. Furthermore, patient inclusion was not consecutive, meaning that not every patient treated during the study period was selected. Selection was based on the availability of materials on the day of the procedure and the patients’ clinical indications.

The study sample therefore consisted of 9 patients scheduled for elective saphenectomy who met the eligibility criteria and consented to RFA treatment on days when an RFA catheter was available, instead of undergoing the traditional saphenectomy procedure. To illustrate the potential cost-effectiveness of RFA, we compared the costs associated with the RFA procedures to those of 9 saphenectomy procedures performed at our institution on the same days as the RFA procedures.

Both the RFA and traditional saphenectomy procedures did not include the removal of collateral veins, non-saphenous varicose veins, reticular veins, or spider veins; either by sclerotherapy or phlebectomy.

Total direct and indirect costs of RFA were calculated as the sum of the respective cost components of each procedure. The cost of surgical consumable items was obtained from the federal government’s price panel.^15^ The patients’ medical records were reviewed for additional data.

Direct costs included materials (e.g., suture, gowns, gloves), medications (e.g., analgesics, antibiotics), anesthesia, operative time, and length of hospital stay. Indirect costs encompassed the time required for patients to return to routine daily activities and days absent from work, according to Brazilian Social Security Administration guidelines. Additional factors that could influence procedural costs were also considered, such as the composition of the surgical team, the presence of interns, and the individual preferences of surgeons and anesthesiologists regarding specific materials and their availability in the hospital.

In Brazil, sick leave is governed by the Brazilian Social Security Administration. Employees who present a medical certificate indicating their inability to work due to illness or injury are entitled to 15 days of sick leave paid directly by their employer. This payment is considered part of the employee’s regular wages and follows the same payment schedule. Beyond 15 days, the responsibility for payment shifts to the social security agency, which provides sickness benefits to the employee for the remainder of their leave, contingent on examination and certification by a social security medical expert. In this scenario, the employer is responsible for ensuring that the employee has completed the necessary paperwork to receive these benefits. In this study, all patients were provided with a medical certificate immediately following their procedure. Patients undergoing RFA received a certificate for 7 days of leave, with the possibility of a 7-day extension. Patients undergoing saphenectomy received a certificate for 30 days of sick leave.

All analyses were descriptive, and no formal statistical testing was performed. All costs are presented in Brazilian *Real* (BRL), with 1 USD = 5.74 BRL (Brazilian Central Bank – US dollar exchange rate on October 1, 2024).

## RESULTS

RFA was successfully performed in all 9 patients. All procedures were performed by a single surgeon (MN), and 13 saphenous veins were ablated in the 9 patients treated. The RFA catheters used in this pilot study were purchased at a mean cost of 1600.00 BRL each.

Table 1 shows the characteristics of all 9 patients treated with RFA, as well as procedural outcomes and associated costs per patient. Patient age ranged from 39 to 65 years, with a mean age of 54 years; 5 patients (55.5%) were men. The mean operating room hourly rate for RFA was 127.50 BRL, while the mean costs of anesthetics/medications, materials, and single-use catheter per patient were 32.63 BRL, 81.49 BRL, and 1600.00 BRL, respectively. This resulted in a total mean cost per procedure of 1841.62 BRL. The mean operative time was 50.89 minutes (range, 35–90 minutes), and the mean length of hospital stay was 7.67 hours. All patients were absent from work for < 15 days (mean, 11.44 days; range, 7–14 days), not incurring sick leave payments from the Social Security Administration.

**Table 1:** Characteristics of each patient treated with radiofrequency ablation, procedural outcomes, and associated costs per patient (n = 9).

**Patient No.**	**No. of saphenous veins treated**	**Age (years)**	**Sex**	**Operative time (min)**	**Length of stay (h)**	**Days absent from work**	**Total cost (BRL)** *	
							**Anesthesia**	**Materials**
1	1	57	F	81	8	14	34.67	69.10
2	2	40	M	40	8	14	34.67	74.98
3	1	60	M	35	8	7	29.72	78.49
4	1	60	F	38	8	12	49.91	76.75
5	1	62	M	40	8	14	21.35	88.30
6	2	62	F	44	8	7	30.91	90.39
7	2	65	M	35	8	14	34.70	73.51
8	2	39	M	55	8	14	21.44	75.80
9	1	41	F	90	5	7	36.35	106.14
Mean	-	54.00	-	50.89	7.67	11.44	32.63	81.49

* Costs are expressed in Brazilian *Real* (BRL) values, where 1 USD = 5.74 BRL (Brazilian Central Bank – US dollar exchange rate on October 1, 2024).

Regarding ultrasound findings, the great saphenous vein was occluded in 2 patients on the left side, in 1 patient on the right side, and in 6 patients bilaterally. All procedures required only 1 vascular surgeon and 1 anesthesiologist in the surgical team. In 6 cases, both local anesthesia and sedation were necessary; in the remaining 3 cases, only local anesthesia was used, and the anesthesiologist did not need to be present.

The characteristics, procedural outcomes, and associated costs of RFA compared to those of traditional saphenectomy are provided in Table 2. The total mean cost of RFA was lower than that of same-day saphenectomy (1841.62 BRL vs 2045.40 BRL).

**Table 2:** Characteristics, procedural outcomes, and associated costs of radiofrequency ablation (RFA) compared to those of saphenectomy.

**Variable**	**RFA (n=9)**	**Saphenectomy (n=9)**
**Demographics**		
Mean age (years)	54.00	56.77
Male sex, n (%)	5 (55%)	5 (55%)
**Outcomes**		
Mean operative time (min)	50.89	190.55
Mean length of stay (h)	7.67	24.00
Mean time off work (days)	11.44	30.00
Surgical team*	1 surgeon + 1 anesthesiologist	2 surgeons + 1 anesthesiologist
**Direct/indirect costs (BRL)**		
Mean cost of RFA catheter	1600.00	0.00
Mean operating room hourly rate	127.50 (150×51 = 7650/60)	455.00 (150×182 = 27300/60)
Mean cost of anesthetics/medications	32.63	34.95
Mean cost of materials	81.49	143.45
Social security-paid sick leave†	0.00	1412.00
Mean total cost	1841.62	2045.40

* RFA was performed with local anesthesia and sedation in 6 cases, and only local anesthesia in 3 cases, resulting in the need for one anesthesiologist per procedure;

† Equivalent to the Brazilian minimum monthly salary in 2024, which denotes government regulation for a minimum monthly rate paid for a worker who works, on average, 44 hours a week for 4 weeks in a month.

Individual patient data for those treated with saphenectomy are available in the Supplementary Material (Table S1).

## DISCUSSION

There is a lack of studies within the Brazilian literature regarding the costs and expenses associated with varicose vein treatment. Given the importance of resource management in our country, it is crucial that we develop expenditure-based approaches to ensure the efficiency and sustainability of the treatments we provide.

Our pilot case series suggests that RFA may offer certain advantages for both patients and hospital managers in the treatment of CVD. For patients, these advantages include use of local anesthesia in most cases and a faster recovery time, allowing for an earlier return to work. For hospital managers, RFA may lead to decreased expenses related to materials, medications, time in the operating room, staffing, and length of hospital stay. Additionally, the reduced time off work for patients treated with RFA (less than 15 days) eliminates the need for sickness benefit payments from the Social Security Administration.

Cost-effectiveness is a crucial consideration in health care, especially as health budgets face increasing constraints.^9^ This necessitates difficult decisions on which interventions to prioritize. When surgery and minimally invasive techniques demonstrate comparable efficacy and safety, cost-effectiveness becomes a decisive factor.^16,17^ In Brazil, studies have evaluated the technical and clinical outcomes of endovenous interventions,^16,18^ but none have assessed the cost-effectiveness of these treatments.

RFA is likely to be a cost-effective treatment option for adult patients with CVD at our institution. We have a substantial backlog of patients awaiting varicose vein treatment, having performed 94 traditional varicose vein surgeries in 2022 and 102 in 2023. The reduced operative time associated with RFA could allow us to treat 400 patients annually at our institution alone. In the city of São Carlos, the current waiting list for varicose vein surgery in the public health system exceeds 600 patients, further highlighting the need for cost-effective solutions.

The initial cost savings of RFA were partially offset by the high unit cost of the catheter (1600.00 BRL), as only 9 units were purchased for the study. However, if RFA is adopted as the preferred treatment, the institution can negotiate reduced prices with manufacturers. This would lower the costs of RFA consumables and further increase the cost-effectiveness of the procedure.

Limitations of this study include the cost data used in our analysis, which are specific to a teaching hospital in Brazil. Additionally, this pilot study was conducted with a small sample size (n = 9) due to the limited availability of RFA catheters, which naturally restricts generalization of the results. If positive results are observed, it will be expanded to a more comprehensive study that includes analysis of clinical outcome data. Selection biases were also identified, primarily due to the limited availability of catheters and the non-consecutive inclusion of patients. To minimize these biases, we took the following methodological precautions: (a) Well-defined clinical criteria – patient selection was based on strict clinical indications, ensuring that only individuals with a clear indication for the procedure were included; and (b) Comparison between techniques – although randomization was not possible, we sought to maintain balance between groups (RFA vs. traditional surgery) to avoid distortions in the results. It is worth noting that assessing clinical outcomes were not an objective of this pilot study, which focused on procedural costs. Based on these preliminary data, we will conduct a follow-up study with a larger sample size to confirm the superiority of RFA over traditional surgery in terms of both procedural costs and clinical outcomes.

## CONCLUSIONS

This pilot study provided essential insights into resource utilization at a Brazilian teaching hospital, with the goal of improving treatment efficiency and ensuring the best cost-benefit ratio for patients. Choosing a less costly, less invasive technique can optimize resources and benefit both patients and hospital managers.

## Supplementary Material

Supplementary material accompanies this paper.

Table S1. Characteristics of patients undergoing saphenectomy and associated costs (n = 9).

This material is available as part of the online article from https://doi.org/10.1590/1677-5449.202401722
