# Supplementary material for: Cost of radiofrequency ablation for chronic venous insufficiency: a pilot study of 9 cases at a teaching hospital in Brazil
Source: J Vasc Bras. 2025 Jul 7;24:e20240172. doi: 10.1590/1677-5449.202401722 (PMC12258812; doi:10.1590/1677-5449.202401722)
Supplement: Table S1. [file jvb-24-e20240172-suppl01.pdf]

Table S1. Characteristics of patients undergoing saphenectomy and associated costs (n = 9).

Patient No.	No. of saphenous veins treated	Age (years)	Sex	Operative time (min)	Length of stay (h)	Days absent from work	Total cost (BRL)*	
							Anesthesia	Materials
1	2	60	M	120	24	30	33.23	197.65
2	1	67	M	120	24	30	33.23	168.63
3	1	49	F	75	24	30	33.23	193.07
4	1	50	M	240	24	30	35.81	152.16
5	1	62	F	180	24	30	35.81	174.44
6	2	41	M	255	24	30	35.81	105.62
7	2	67	F	260	24	30	35.81	66.02
8	1	56	M	225	24	30	35.81	104.41
9	2	59	F	240	24	30	35.81	129.08
Mean	-	56.77	-	190.55	24	30	34.95	143.45

* Costs are expressed in Brazilian *Real* (BRL) values, where 1 USD = 5.74 BRL (Brazilian Central Bank – October 1, 2024).
